# Ayurvedic herbal formulations Haridra Khanda and Manjisthadi Kwath (brihat) in the management of allergic rhinitis: A pharmacological study

**DOI:** 10.1016/j.heliyon.2024.e31937

**Published:** 2024-05-28

**Authors:** Rudranil Bhowmik, Md Adil Shaharyar, Mahibub Mahamadsa Kanakal, Arnab Sarkar, Syeda Ayesha Farhana, Shalam M. Hussain, Abdullah Khan, Pallab Mandal, S. Roshan, Achintya Mitra, Sanmoy Karmakar

**Affiliations:** a Bioequivalence Study Centre, Department of Pharmaceutical Technology, Jadavpur University, Kolkata, 700032, India; b Faculty of Pharmacy, Quest International University, Ipoh, Perak, Malaysia; c Department of Pharmaceutics, College of Pharmacy, Qassim University, Buraidah, Qassim, 51452, Saudi Arabia; d Department of Clinical Pharmacy, College of Health Sciences and Nursing, Al-Rayan Colleges, AL-Madinah, AL-Munawarah, 20012, Saudi Arabia; e Deccan School of Pharmacy, Osmania University, Hyderabad, 500001, Telangana, India; f Regional Ayurveda Research Institute, Central Council for Research in Ayurvedic Sciences, Ministry of AYUSH, Govt. of India, Ranikhet, Almora, Uttarakhand, India

**Keywords:** Polyherbal drug formulations, Histamine & H1 receptor, Mast cell, Intracellular Calcium, Allergy, *in vivo* study

## Abstract

This study aims to pharmacologically validate Haridra Khanda (HK) and Manjishthadi Kwatham (brihat) (MMK) in allergy management using *invivo* and *invitro* studies to rationalize the prescription of these two ayurvedic polyherbal drug formulations, which are currently used in Indian government hospitals. Experimental animals received HK and MMK orally from day 0 to day 14 and histamine (1 mg/kg b.w/i.v) and 1 % evans blue (EB) (0.1 mL) via tail vein on day 14. The compound 48/80 (intracutaneous) challenged mice model followed the same technique. The former mimicked acute anaphylaxis and the latter mast cell degranulation. For both models, EB dye leakage was quantified spectrophotometrically to determine vascular permeability. Plasma histamine was measured in Compound 48/80-induced animals using LC-ESI-MS/MS. The guineapig received HK and MMK p.o. and 0.6 % histamine sprayed in a histamine chamber to simulate allergic rhinitis. Blood eosinophil count and sneeze rate were measured in histamine-challenged guineapigs. Goat R.B.C. membrane stability assay (mammalian cell membrane toxicity) and intracellular histamine-induced cytosolic Ca^2+^ release assay in Chinese hamster ovary (CHO) cells were performed *in vitro*. For both histamine and Compound 48/80 challenged animals, HK (22.81 % and 14.58 %) and MMK (19.71 % and 22.40 %) significantly reduced EB dye leakage (p < 0.05). Both formulations, HK and MMK considerably (p < 0.05) decreased plasma histamine (29.62 % and 25.37 % respectively) in mice and eosinophilic count (11.56 % and 9.94 % respectively) and sneeze rate (42.58 % and 29.03 % respectively) in guinea pigs. In membrane stability experiment, HK and MMK reduced RBC lysis. Both HK and MMK raw/dialysate blocked CHO cell cytosolic Ca^2+^ release. HK and MMK activities mimic mast cell stabilization with possible H1 receptor inactivation seen by decreased Ca^2+^ efflux and thus indicate potential for allergic rhinitis management. The combination of activities is usually related with curative and prophylactic therapy and might lead future clinical trials and therapies.

## Graphical abstract

**Figure:**
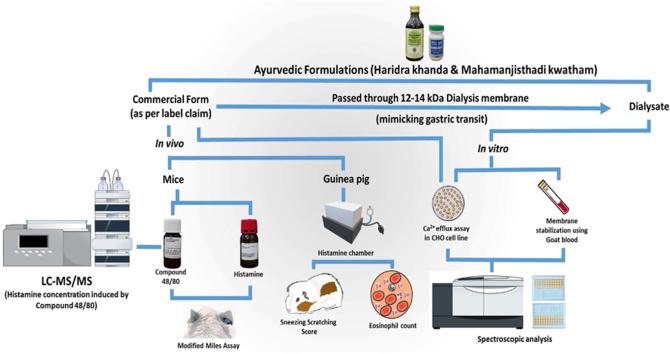

## 1 Introduction

Although allergy disorders including asthma and allergic rhinitis have become more common over the past ten years, the precise reasons are still unknown. The increased prevalence of atopic disease has been attributed to both air pollution and dietary habit changes. Seasonal allergic rhinitis, asthma, atopic dermatitis, urticaria and itching are various forms of allergy disorders [1]. About 20 % of people experience acute urticaria throughout their lifetime. In a year, a mere 0.08 % of the population from every major region of the United States experienced chronic urticaria. However, this rate drastically increased in european countries, ranging from 0.38 % to 0.8 %. Notably, adolescents in China have a much higher prevalence of 2.7 % as revealed by a thorough cross-sectional investigation [2].

Itching is a common symptom observed in dermatology outpatient clinics across Europe. More than half (54.4 %) of the adult patients in these clinics experience itching, compared to just 8 % in the control group. The severity of itching is particularly high in patients diagnosed with prurigo. This symptom is not limited to hospital settings only; it is also common in private dermatology practices. In another private practice based study in Germany, over a third (36.2 %) of the patients reported itching over a one-week period, where the majority (87.6 %) of these cases were chronic in nature [3]. Children typically have a higher prevalence of atopic dermatitis than adults do, according to data on the incidence and prevalence of the condition. Abuabara et al. conducted a study in the UK to determine the prevalence of Atopic Dermatitis among 8,604,333 UK residents between 1994 and 2013. 12.3 % of children aged 17 and under, 5.1 % of people aged 18 to 74, and 8.7 % of individuals over 75 were reported to have the said condition [4]. In both the United States and Europe, 20–30 % of adults and possibly a somewhat greater number of children suffer from allergic rhinitis [5].

Despite advances in our knowledge of the pathogenesis of allergic diseases and the discovery of synthetic molecules, the prevalence of allergic rhinitis, asthma, atopic dermatitis, urticaria and itching has increased as evident from the prevalence data. WHO has recognized Ayurveda globally, addressing the issue of standardization of traditional medical systems in both academics and healthcare systems. Furthermore, WHO reports that 65 % of the global population predominantly depends on traditional medicines for their healthcare. Some multiple biological pathways or factors are involved in a disease. So, it is quite rational to use multiple components/herbs to target multiple biological targets [6,7].

The Sarangdhar Samhita, an Ayurvedic text, promoted the use of many herbs to improve the effectiveness of treatments. The active phytochemical components in individual plants are not enough to provide the intended therapeutic effects. Combining different herbs in a specific ratio improves the therapeutic impact and lessens the toxicity. Ayurveda finds its root origin in India and involves the concept of 3 basic balanced doshas and thus health is attained, but imbalance results in disease. An individual's Prakriti is identified based on these Panchamahabhutas and Tridosha, and a specific treatment regimen can be suggested by their particular constitution [8,9]. The traditional Acharyas (traditional Ayurvedic practitioners) of India formulated traditional ayurvedic polyherbal drug formulations to address the various manifestations of allergy. The practice of these traditional formulations has been passed from one generation as a traditional practice.

Haridra Khanda (HK) and Manjishthadi Kwatham (brihat) (MMK) are two ayurvedic polyherbal drug formulations that are frequently prescribed by ayurvedic practitioners for ages in and around India. The constituents of HK and MMK are summarized in Table 1, Table 2, respectively. In the ayurvedic system of medicine, HK has been used in the management of urticaria, itching and blister while MMK in gout, disease of the skin, facial palsy, a disorder of adipose tissue and eye [10,11]. HK is a bright yellow colored powdered solid dosage form while MMK is a dark brown colored tablet. This present research work aims to pharmacologically validate Haridra Khanda (HK) and Manjishthadi Kwatham (brihat) (MMK) in the management of allergies involving *invivo* and *invitro* studies to form a rational basis for the prescription of these two ayurvedic polyherbal drug formulations, currently in clinical use in Indian government hospitals.

**Table 1:** Constituents of Haridra Khanda as per its label claim.

Plant name (Scientific name)	Part used	Amount present per 10 (g)
Haridra (*Curcuma longa*)	Rhizome	2.1 gm
Shunti (*Zingiber officinale*)	Rhizome	150 mg
Maricha (*Piper nigrum*)	Fruit	150 mg
Pippali (*Piper longum*)	Fruit	150 mg
Twak (*Cinnamum zeylanicum*)	Stem Bark	150 mg
Ela (*Elettaria cardamomum*)	Seed	150 mg
Patra (*Cinnamomum tamala*)	Leaf	150 mg
Haritaki (*Terminalia chebula*)	Pericarp	150 mg
Vibhitaki (*Terminalia bellerica*)	Pericarp	150 mg
Amalaki (*Emblica offinalis)*	Pericarp	150 mg
Nagakeshar (*Mesua ferrea*)	Stamens	150 mg
Musta (*Cyperus rotundus*)	Root Tumber	150 mg
Vidanga (*Embelica ribes*)	Fruit	150 mg
Trivrat (*Operculina turpethum*)	Root	150 mg
Lauha (incinerated iron)	–	150 mg
Khanda (sugar)	–	7.5 gm
Sodium benzoate (Preservative)	–	q.s.

**Table 2:** Constituents of Manjishthadi Kwatham (Brihat) as per its label claim.

Plant name (Scientific name)	Part used	Amount present per tablet
Manjistha *(Rubia cordifolia)*	Root Tuber	0.394 g
Kutaja *(Holarrhena pubescens)*	Stem Bark	0.394 g
Amrita *(Tinospora cordifolia)*	Stem	0.394 g
Khana *(Cyperus rotundus)*	Root Tuber	0.394 g
Bala (*Sida cordifolia*)		0.394 g
Vacha/shatgrandha *(Acorus calamas)*	Root	0.394 g
Sunthi *(Zingiber officinale)*	Rhizome	0.394 g
Haridra *(Curcuma longa)*	Rhizome	0.394 g
Daruharidra *(Berberis aristate)*	Root	0.394 g
Aristha *(Azadirachta indica)*	Stem Bark	0.394 g
Patola mula *(Trichosanthes cucumerina)*	Root	0.394 g
Katuka *(Neopicrorhiza scrophulariiflora)*	Root	0.394 g
Bharngi (*Clerodendrum serratum*)	Root	0.394 g
Agni (*Plumbago zeylanica*)	Root	0.394 g
Vidanga (*Embelia ribes)*	Seed	0.394 g
Murva (*Chonemorpha fragrans)*	Root	0.394 g
Daru (*Cedrus deodara)*	Root	0.394 g
Bhringa (*Eclipta prostrata)*	Plant (whole)	0.394 g
Magadha (*Piper longum)*	Fruit	0.394 g
Trayanti (*Gentiana kurroo)*	Root	0.394 g
Patha (*Cyclea peltate)*	plant (whole)	0.394 g
Sathi (kaempferia galanga)	Underground stem	0.394 g
Gayatri (*Acacia catechu)*	Root	0.394 g
Pathya (*Terminalia chebula)*	Heart Wood	0.394 g
Dhatri (*Phyllanthus emblica)*	Fruit Rind	0.394 g
Vibhitakai (*Terminalia bellirica)*	Fruit Rind	0.394 g
Kirataka (*Swertia chirayita)*	Fruit Rind	0.394 g
Mahanimba *(Melia azedarach)*	Root	0.394 g
Asana *(Pterocarpus marsupium)*	Stem Bark	0.394 g
Aragwadha *(Cassia fistula)*	Heart Wood	0.394 g
Syama *(Operculina turpethum)*	Stem Bark	0.394 g
Avalguja *(Cullen corylifolium)*	Root	0.394 g
Chandana *(Santalum album)*	Seed	0.394 g
Varanaka *(Crataeva magna)*	Heart Wood	0.394 g
Puthika (oldenlandia corymbosa)	Plant (whole)	0.394 g
Danti *(Balospermum mondanum)*	Root	0.394 g
Sakhotaka *(Streblus asper)*	Stem Bark	0.394 g
Vasa *(Justicia beddomei)*	Root	0.394 g
Parpata *(Hedyotis corymbose)*	Root	0.394 g
Sariba *(Hemidesmus indicus)*	Plant (whole)	0.394 g
Krishnasariba (*Ichnocarpus frutescens*)	Root	
visha *(Aconitum heterophyllum)*	Root	0.394 g
Ananta *(Tragia involucrate)*	Root	0.394 g
Vishala *(Citrullus colocynthis)*	Root	0.394 g
Jala *(Plectranthus vettiveroides)*	Plant (whole)	0.394 g
Jastimadhu (*Glycyrrhiza glabra*)	Root	0.394 g
Magalyapushpi (*Clitoria ternatea*)	Roots	0.394 g

## 2 Materials and methods

### 2.1 Chemicals

HK (Batch No.RJA21094, mfg by Revinto Lifescience Pvt.Ltd, Lic No. AUS-715) and MMK (Batch No.526994, manufactured by Arya baidya Sala, Kottakkal, Lic No.45/25D87) were donated by Central Ayurveda Research Institute for Drug Development, Kolkata. Dialysis membrane-70 (catalogue no. LA393, Himedia), 10X Phosphate Buffered Saline (PBS) (cat no. 78529, SRL), goat blood, standard Pheniramine maleate (cas.no.132-20-7, Merck), Evans Blue (Cas.no.314-13-6, Sigma Aldrich), formamide (Cas.no.75-12-7, Merck), Histamine (Cas.no. 56-92-8, Sigma Aldrich), Compound 48/80 (C 48/80) (Cat.no. C2313, Merck), ENG Scientific Eosin Y, 1 % aqueous Solution (Cat.no. ES8901), Acetone (Fisher Chemical™, Cat.no. A18-4), CD CHO Medium (Cat.no. 10743029, Gibco, ThermoFisher Scientific), Fluo-4 NW Calcium Assay Kit (Cat.no. F36206, ThermoFisher Scientific, USA), Hanks' Balanced Salt Solution (HBSS) without Ca^2+^, Mg^2+^ for Pierce™ Primary Cell Isolation Kits (Cat.no. 88284, ThermoFisher Scientific), Trypsin-EDTA (0.05 %), phenol red, (Cat.no. 25300062, Gibco, ThermoFisher Scientific), DAPI (Cat.no. D3571, ThermoFisher Scientific), Metoprolol (Cas no. - 56392-17-7, Merck).

**Equipments:** magnetic stirrer (Remi 2MLH), Histamine chamber (Scientific Instrument Traders, Ambala, India), Bright field microscope (Olympus CH20i, Japan), Neubauer chamber (Superior, Marienfeld, Germany), 0.22 μm filter (Millipore®; Thermo Fisher Scientific, Waltham, MA, USA), SpectraMax (M5 Series Multi-Mode Microplate Readers, Molecular Devices, LLC, USA), flat black clear bottom 96 well plates (Cat.no. 266120, ThermoFisher Scientific, USA), Zeiss LSM 700 Confocal microscope (Carl Zeiss 700, Germany), Ab Sciex QTRAP (API-4000).

### 2.2 Animals

Swiss albino mice (20–25 g) and guineapigs (350–400g) were utilised in these experiments where n = 6 was assigned for each group. The mice were maintained under a 12 h cycle of light and dark phase, at a temperature of 25 ± 2 °C with accessible food and drinking water. All the involved animal experiments were approved by the Institutional Animal Ethics Committee (IAEC) of Dept. of Pharmaceutical Technology, Jadavpur University, through project proposal no. JU/IAEC-22/21.

### 2.3 Histamine-challenged mice (n = 6)

Control_*H*_ group: Saline water administered p.o. for 14 days.

Disease Control_*H*_ group: Saline water (p.o.) administered for 14 days.

HK_*H*_ group: 1.38 g/kg/day (p.o.) HK administered concurrently for 14 days.

MMK_*H*_ group: 0.25 g/kg/day MMK (p.o.) administered for 14 days.

Standard_*H*_ group: [5.85 mg/kg (i.p.) Pheniramine maleate injected on the 14th day before histamine administration].

0.1 mL of sterile 1 % EB dye and 1 mg/kg b.w. of histamine was injected via tail vein on the 14th day in all the groups (Sparing the control_H_ for histamine administration) [12].

### 2.4 Compound 48/80 challenged mice (n = 6)

Control_48/80_: Saline water p.o. for 14 days.

Disease Control_48/80_ group: Saline water p.o. for 14 days.

HK _48/80_ group: 1.38 g/kg/day HK p.o. for 14 days.

MMK_48/80_ group: 0.25 g/kg/day MMK for 14 days.

Standard_48/80_ group: Sodium cromoglycate 80 mg/kg/i.p. for 14 days.

0.1 mL of sterile 1 % EB dye (via tail vein) and 3 μg C 48/80 in 10 μl saline administered intracutaneously on the 14th day in all the groups (Sparing the control_H_ for C 48/80 administration) [13,14].

### 2.5 Guineapig model (n = 6)

Control_GH_ group: Saline water p.o. for 7 days.

Disease controlGH group: Saline water p.o. for 7 days.

HKGH group: 335 mg/kg/day HK for 7 days.

MMKGH group: 32.1 mg/kg/day MMK for 7 days.

StandardGH group: 3.25 mg/kg/day i.p. Pheniramine maleate for 7 days.

In the Guineapig model, guineapigs belonging to all the groups except control_GH_ group was sprayed with 0.6 % histamine on the 7th day in the histamine chamber. The animals in the control_GH_ group was sprayed with water only [15].

### 2.6 Sneezing rate in guineapig

In a Histamine chamber, male guinea pigs weighing between 350 and 450 g were exposed to a 0.6 % histamine dihydrochloride aerosol created by compressed air at a pressure of 4 atm. For 20 min, the animals were exposed to histamine 10 times or 10 sprays. Sneezing was characterized by an explosive expiration just after a deep inspiration. Sneezing was observed and recorded and number was noted [15].

### 2.7 Blood collection for eosinophilic count

Blood from guinea-pig belonging to the histamine chamber was collected from the saphenous vein. The quantification of eosinophils was accomplished by combining 10 μl of blood with 90 μl of Discombe's solution, followed by the counting of cells with stained granules using a haemocytometer after a 5 min interval. Formula for counting eosinophils in Haemocytometer: (cell count*dilution factor)/(Area counted*depth). Preparation of Discombe's solution was carried out by mixing 1 % aqueous eosin Y (5 vol) with 5 vol of acetone and distilled water 90 vol [16,17].

### 2.8 Preparation of dialysate from the crude formulation

Dialysate of MMK and HK was prepared using the dialysis bag method. Samples of 750 mg of MMK and 1000 mg of HK were dissolved in 1500 μl and 2000 μl of water respectively. Samples were added by putting them in a sealed dialysis bag with a molecular weight cutoff (MWCO) of 12–14 kDa. After being submerged in 100 mL of PBS with a pH of 7.4 and subjected to incubation at a temperature of 37 °C. The incubation process included placing the bags on a magnetic stirrer set at a speed of 100 rpm. After 24 h, the samples were collected and filtered using 0.22 μm filters [18].

### 2.9 Preparation and application of Evans blue (Miles assay) in mice

EB dye (0.1 mL of 1 % w/v in 0.9 % saline) was prepared and Sterilized by autoclaving (121 °C, 15 psig for 15 min) followed by passing the solutions through a 0.22 μm filter aseptically. 100 μl of EB solution was injected into the tail vein of mice and allowed the dye to properly circulate for 30 min. A cervical dislocation was used to kill the mice 30 min after the challenge, and ear samples were taken. EB was extracted for 24 h at 55 °C in 250 μl of formamide, and its concentration was measured in a spectrophotometer at 620 nm [19].

### 2.10 Preparation and application of histamine and compound 48/80 in mice

Filtered (0.22 μm) histamine solution was injected in mice intravenously in a dose of 1 mg/kg b.w. Each animal was given 100 μl of the histamine solution prepared in 0.9 % saline. We found that administration of 0.2 mmol/kg body weight b.w. of dose was not tolerated in our experimental mice leading to high mortality rate. So we adjusted the dose to 1 mg/kg b.w. where hyperpermeability was observed without animal death [20].

Similarly, C 48/80 was dissolved in 0.9 % saline. The prepared C 48/80 (3 μg in 10 μl saline) was filtered using 0.22 μm filter [13].

### 2.11 Membrane stability assay

Blood was taken from a healthy goat that had not had any pharmaceutical treatment 14 days prior to this study. Sodium oxalate was used as an anticoagulant in the blood. Furthermore, maintaining all of the blood samples at 4 °C for 24 h was ideal for preservation before use. After undergoing a 5 min centrifugation at 2700 rpm, the supernatant was discarded. With an autoclaved isotonic saline solution, the cell suspension was cleaned. The same centrifugation conditions were used again till the supernatant was clear and visually colourless. The cells were suspended again in 20 % (v/v) PBS solution (10 mM, pH 7.4).

A solution was prepared by combining 0.5 mL of 20 % RBC suspension, 1 mL of 0.15 M PBS (pH 7.4), and 2 mL of 0.25 % sodium chloride. The mixture was mixed and then allowed to stand at room temperature for 15 min 0.5 mL of crude extracts of formulations with concentrations of 250 μg/mL, 500 μg/mL, and 1000 μg/mL were added. Aspirin was utilised as a standard agent at an equivalent concentration to the extracts. Following incubation at room temperature, all experimental samples were centrifuged at 5000 rpm for 5 min, and absorbance was measured at 530 nm [21].

### 2.12 Plasma collection and quantification of plasma histamine in compound 48/80 challenged mice

From the retro-orbital plexus of mice, blood samples of less than 1 mL were collected and plasma was extracted by cold centrifugation (4 °C) at 3500 rpm for 10 min. Histamine was quantified in the plasma using LC-MS/MS. To prepare a 100-fold concentrated stock solution, 1 mL of deionized water was added to the bottle containing the powdered protease-inhibitor cocktail. 100 mL of (1:100) protease inhibitor solution was prepared by diluting the buffer. After centrifugation, the obtained plasma was rapidly mixed with 10 μL of the protease inhibitor solution per sample and eventually stored at −20 °C.

## 3 Methodology for LC-MS/MS study

Utilising LC-MS/MS triple-quadruple quantification facilitates the precise detection of minimal changes in blood histamine levels or other endogenous substances post-test substance administration, enabling data collection with a very low limit of quantification. We utilised a thermostatic auto sampler, on-line degasser, and binary pump all made by Shimadzu with an LC system (LC-20AD). An Ab Sciex QTRAP (API-4000) mass spectrometer with an ESI (electrospray ionization) source was linked to the LC. The data was collected using Analyst Software version 1.6.3. For separating the histamine Phenomenex Kinetex (5μ C18 100A 50 × 3 mm) column was employed. The chromatographic conditions include 10 μl injection volume at a flow rate of 0.5 mL/min and a column temperature of 25 °C, a linear gradient was eluted using mobile phases A and B. ESI with both negative and positive ions were used as the ionization source. Nitrogen was used as curtain gas and was kept at a flow of 20.00 psig while GS1 and GS2 gas were kept at 40.00 and 45.00 psig, respectively. Both the temperature of the gas and capillary voltage was set at 400 °C and −4500.00 V respectively. Similarly, the declustering potential (DP) and the collision energy was applied for parent ionization and product ionization respectively. Multiple reactions monitoring (MRM) was the last step in the mass spectrometry detection response. Liquid-liquid extraction method was used for extracting histamine from plasma matrix where metoprolol was used as an internal standard (IS) [22].

### 3.1 Quantification of intracellular Ca^2+^ signaling in CHO cells

Calcium signaling in CHO cells was quantified using Fluo-4 NW Calcium Assay Kit. The calcium response rate was assessed according to the kit manual with minor modifications in the procedure reported earlier [23,24]. In brief, CHO cells were cultured in flat black clear bottom 96 well plates at a density of 2000 cells per well in CD CHO media. All the solutions were pre-incubated at 37 °C before supplementing them to the cells. After reaching 70 % confluence, cells were pre-incubated with vehicle control or at different concentrations of raw and dialysate of MMK and HK, for 16 h and then exposed to Fluo4 NW solution for 60 min at room temperature. The medium was aspirated from the culture and cells and was washed twice with HBSS assay buffer. Cells were cultured in assay buffer and then stimulated with histamine (10 μM) at different incubation durations for a maximum of 120 min. The fluorescence intensity in each well was measured at an excitation wavelength of 494 nm and an emission wavelength of 516 nm. The peak response was recorded right after washing and at 20min intervals up to 120min. The highest intensities of each sample were adjusted in relation to the background and the average reaction of 10 μM histamine (100 %). The values were plotted against the time elapsed between eliminating unbound antihistamines from the cells and activating the cells with histamine. The fluorescence images of cells at different washout durations were documented using a Zeiss LSM 700 Confocal microscope (Carl Zeiss 700, Germany) at 20× magnification after mounting with DAPI solution.

### 3.2 Statistical analysis

Data analysis was conducted with GraphPad Prism® 9.0 software (GraphPad Software Inc., La Jolla, CA, USA). All the data were expressed as Mean ± SD and graphically represented using bar diagram. The one-way ANOVA test followed by Tukey's test was used to evaluate the statistical comparisons between different groups. A comparison was considered statistically significant when p ≤ 0.05.

## 4 Result

### 4.1 HK and MMK attenuates histamine and compound 48/80 induced EB dye leakage

**Fig. 1:**
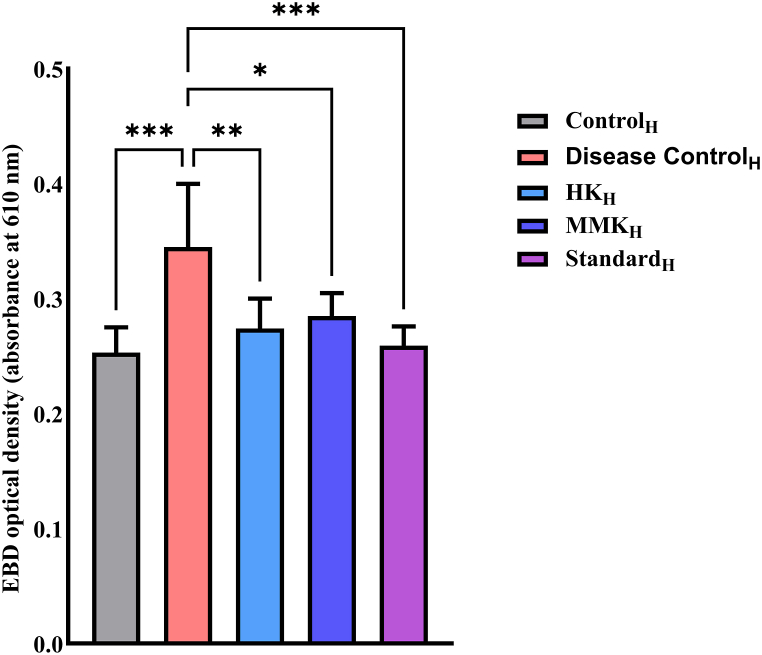
Represents the optical density of EB dye leakage of control_H_, disease control_H_, HK_H_, MMK_H_ and Standard_H_ denoted groups in mice model on administering histamine (i.v.) externally where *p < 0.05, **p < 0.01, ***p < 0.001. The percentage inhibition of HK, MMK and standard (pheneriamine maleate) were found to be 22.81 %, 19.71 % and 27.04 % respectively. Statistical comparisons made are as follows; control_H_ group vs disease control_H_ group; disease control_H_ Vs HK_H_, MMK_H_ and Standard_H_.

**Fig. 2:**
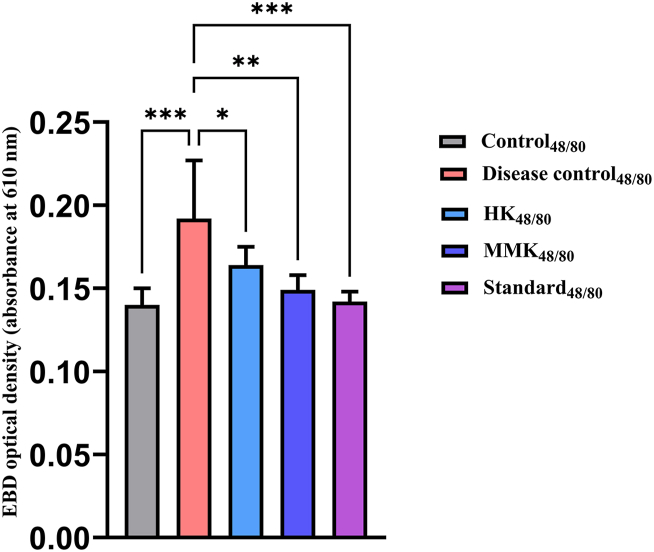
Represents the optical density of EB dye leakage of control_48/80_, disease control_48/80_, HK_48/80_, MMK_48/80_ and Standard_48/80_ denoted groups in mice model on administering C 48/80 where * p < 0.05,**p < 0.01, ***p < 0.001. The percentage inhibition of HK, MMK and standard (sodium chromoglycate) were found to be 14.58 %, 22.40 % and 26.04 % respectively. Statistical comparisons made are as follows Control_48/80_ vs disease control_48/80_ and disease control_48/80_ vs HK_48/80_, MMK_48/80_ and Standard_48/80_.

**Fig. 3:**
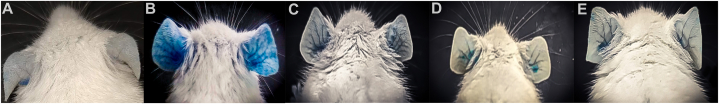
**Represents** the image of the two ears of the mice belonging to different groups showing EB dye leakage where (A) control_H_ (B) disease control_H_ (C) MMK_H_ (D) HK_H_, and (E) Standard_H_ groups in mice model on administering histamine externally (i.v.).

The intensity of EB dye leakage from mice ears, was assessed through two methods: histamine and compound 48/80 induced EB dye leakage. In the histamine and compound 48/80 induced model significant EB dye leakage was observed in the disease control group compared to the control group (***p < 0.001) of both the models. In the histamine model, treatment with HK, MMK and the standard drug significantly attenuated dye leakage (**p < 0.01, *p < 0.05, and ***p < 0.001, respectively) as compared to the disease control_H_ group. From the observed Fig. 1, it can be derived that HK showed better attenuation of dye leakage than MMK. Similarly, in the Compound 48/80-induced model treatment with HK, MMK and the standard drug significantly attenuated dye leakage (*p < 0.05, **p < 0.01, and ***p < 0.001, respectively) as compared to the disease control_48/80_ group. From our observation of Fig. 2 it can be interpreted that MMK prevented the dye leakage more than HK. The extravasation of EB dye from the mice ear, as observed in this specific investigation, is depicted in Fig. 3.

### 4.2 HK and MMK reduces sneezing in guineapigs

**Fig. 4:**
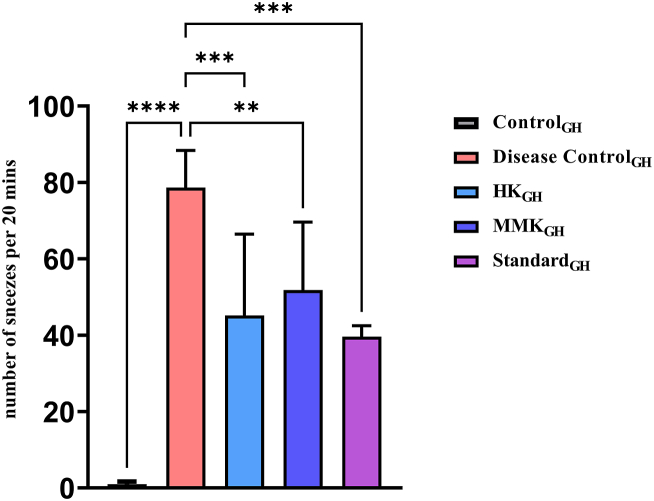
Represents number of sneezes per 20 min in guineapig evaluated in a histamine chamber. The statistical comparisons were made as follows Control_GH_ vs disease control_GH_ and disease control_GH_ vs HK_GH_, MMK_GH_ and Standard_GH_ where * p < 0.05, **p < 0.01, ***p < 0.001, ****p < 0.0001. The percentage inhibition of HK, MMK and standard (sodium chromoglycate) were found to be 42.58 %, 34.11 % and 49.6 % respectively.

The number of sneezes per 20 min in guineapig is represented by Fig. 4. There was a highly significant (****p < 0.0001) increase in the disease control_GH_ as compared to the Control_GH._ The number of sneezes in all the three groups namely HK_GH_ (***p < 0.001), MMK_GH_ (**p < 0.01) and standard_GH_ group (***p < 0.001) decreased significantly as compared to the disease control_GH_ group. Though, in both the HK and MMK group, number of sneezes decreased but the number of sneezes was lesser in the HK_GH_ group than the MMK group animals.

### 4.3 HK and MMK attenuates blood eosinophilic count in guineapigs

**Fig. 5:**
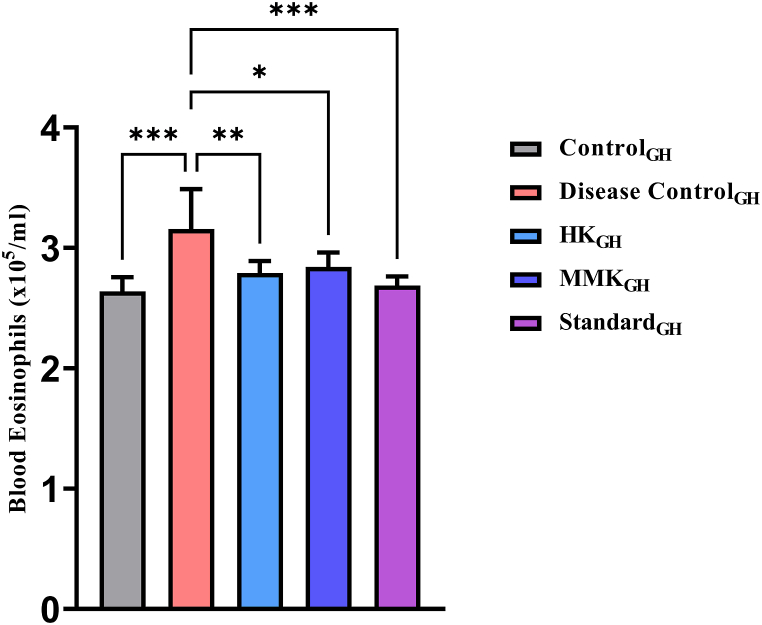
Represents blood eosinophilic count in guineapigs evaluated in neubauer Chamber. Statistical comparison was made as follows control_GH_ vs disease control_GH_ and disease control_GH_ vs HK_GH_, MMK_GH_ and standard_GH_ where * p < 0.05,**p < 0.01, ***p < 0.001. The percentage inhibition of HK, MMK and standard (sodium chromoglycate) were found to be 11.56 %, 10.04 % and 14.82 % respectively.

Fig. 5 represents blood eosinophilic count measured on the 7th day of the experiment in guineapigs. Guineapigs in the disease control_GH_ group showed significant elevation (***p < 0.001) of blood eosinophil count as compared to the control_GH_ group. The blood eosinoophilic count in all the groups namely HK_GH_ (**p < 0.01), MMK_GH_ (*p < 0.01) and standard_GH_ group (***p < 0.001) was significantly reduced as compared to the disease control_GH_. From our observation of Fig. 5, the HK_GH_ group animals showed more decrease in blood eosinophilic count than the MMK_GH_ group.

### 4.4 HK and MMK attenuates plasma histamine in compound 48/80 induced mice

**Fig. 6:**
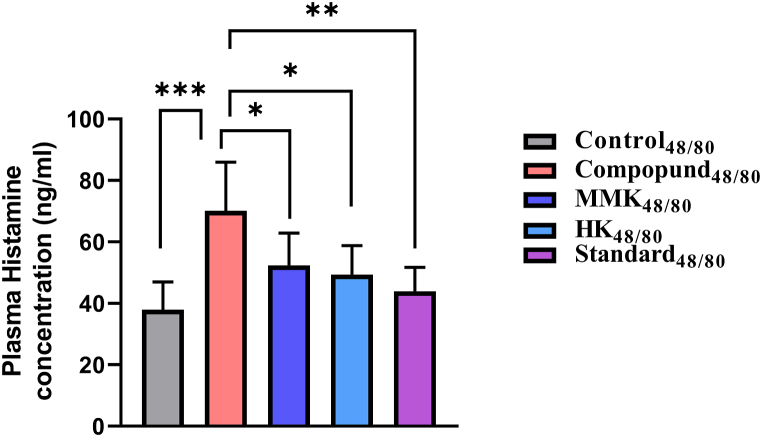
Represents plasma histamine in C 48/80 challenged mice evaluated using LC-MS/MS. The statistical comparison was made as follows control_48/80_ vs disease control_48/80_, disease control_48/80_ vs HK_48/80_, MMK_48/80_ and Standard_48/80_ where * p < 0.05,**p < 0.01, ***p < 0.001. The percentage inhibition of HK, MMK and standard (sodium chromoglycate) were found to be 29.62 %, 25.37 % and 37.28 % respectively.

Fig. 6 represents the plasma histamine level in C 48/80 induced mice model measured by LC-ESI-MS/MS. The plasma histamine level in the **Disease Control**_**48/80**_ group significantly increased (***p < 0.001) in comparison to the **Control**_**48/80**_ group animals. The plasma histamine level significantly decreased in **HK group**_**48/80**_ (*p < 0.05), **MMK Group**_**48/80**_ (*p < 0.05) and **Standard group**_**48/80**_ (**p < 0.01) when compared to the **Disease Control**_**48/80**_ group. From the observed Fig. 6, the plasma histamine in the **HK group**_**48/80**_ group was found to be lesser than **MMK Group**_**48/80**_ group animals.

### 4.5 Protective effect on the RBC In vitro by HK and MMK

Fig. 8 represents the anti-inflammatory activity of HK and MMK by induction of hypotonicity in goat RBC. The anti-inflammatory activity of HK and MMK is expressed as inhibition of RBC haemolysis in percentage. The anti-inflammatory effect is the extent of giving protection to goat RBC by the different concentrations of HK and MMK in comparison to the standard drug aspirin. At 1000 μg/mL concentration, HK (94.33 %) and MMK (92.67 %) showed the highest % inhibition of RBC membrane rupture.

**Fig. 7:**
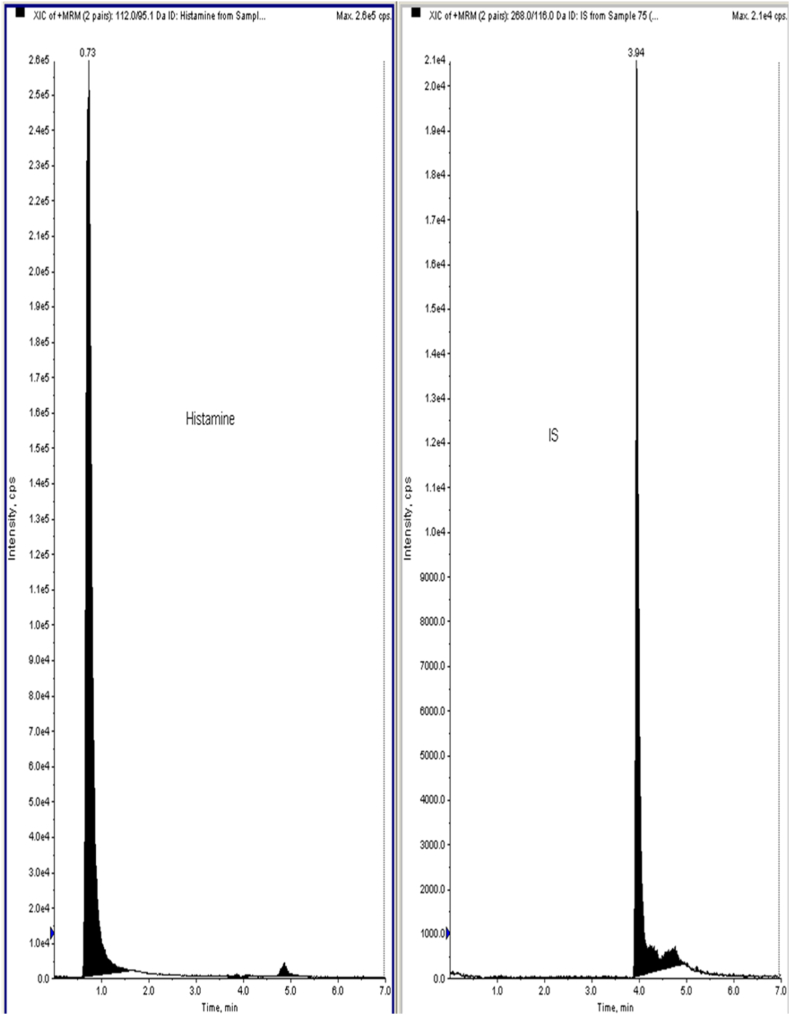
Peaks of raw data obtained from an LC-MS/MS based MRM of plasma Histamine with metoprolol as an internal standard (IS).

**Fig. 8:**
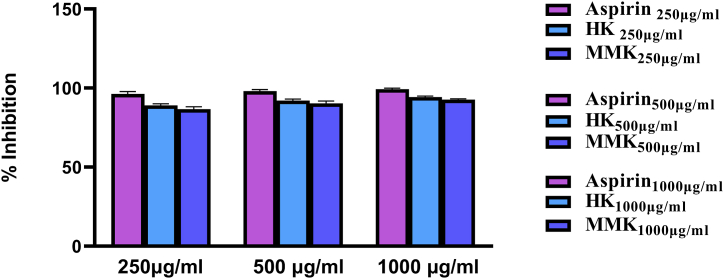
represents protection of RBC in terms of % inhibition of standard drug (aspirin), HK and MMK's (ability to prevent RBC rupture) at concentrations of 250,500 and 1000 μg/mL.

### 4.6 HK and MMK inhibits intracellular calcium release in CHO cells

**Fig. 9:**
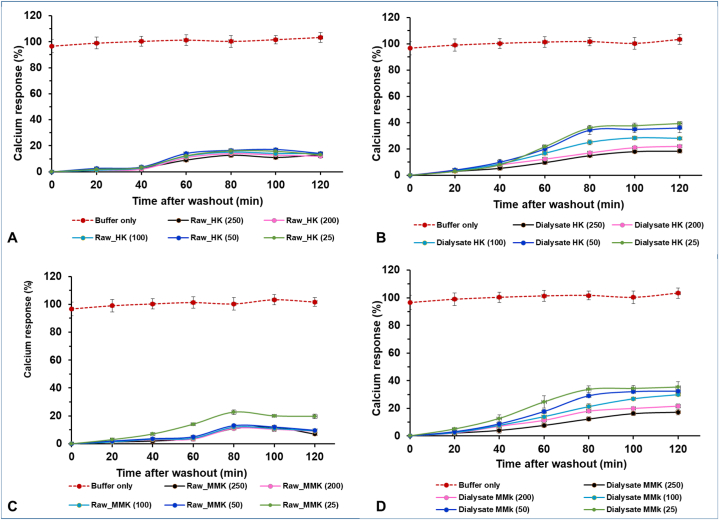
Represents calcium response % vs Time after washout (mins) for different concentrations of HK raw, HK dialysate, MMK raw and MMK dialysate in CHO cell line. The red colored line denotes the calcium response % elicited by buffer. All concentrations are mentioned in μg/ml in case of raw HK and MMK while their dialysates are in μg/ml equivalent. The varying color indicates different concentrations of HK and MMK in their raw and dialysate forms.

Fig. 9 represents calcium response % vs time after washout (mins) in CHO cells. In Fig. 9A i.e. for HK raw, the calcium response % started to increase steeply after the 40th min but did not change much from the 60th min till the 120th min. HK raw at a concentration of 250 μg/ml showed the best intracellular cytosolic calcium inhibition against histamine challenge in CHO cells. For HK's dialysate i.e. Fig. 9B, the calcium response % started to increase from the 20th min washout period. Among the different concentrations of HK dialysate, 250 μg/ml equivalent showed the best inhibition of calcium response %.

**Fig. 10:**
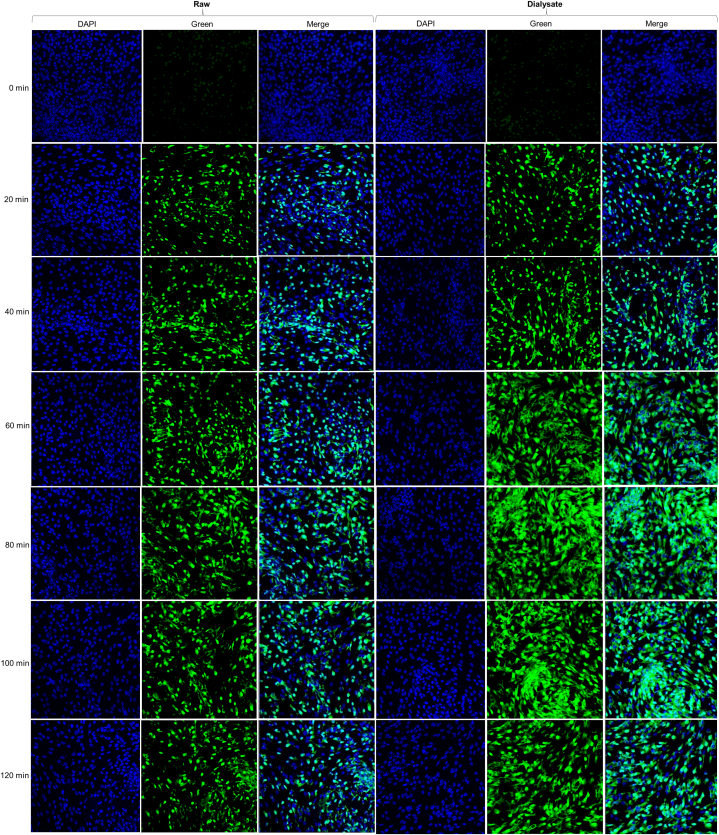
Represents the fluorescence images of CHO cells at different washout durations for 250 μg/mL of HK raw and 250 μg/mL equivalent of HK dialysate is shown using Confocal microscopy at 20× magnification.

**Fig. 11:**
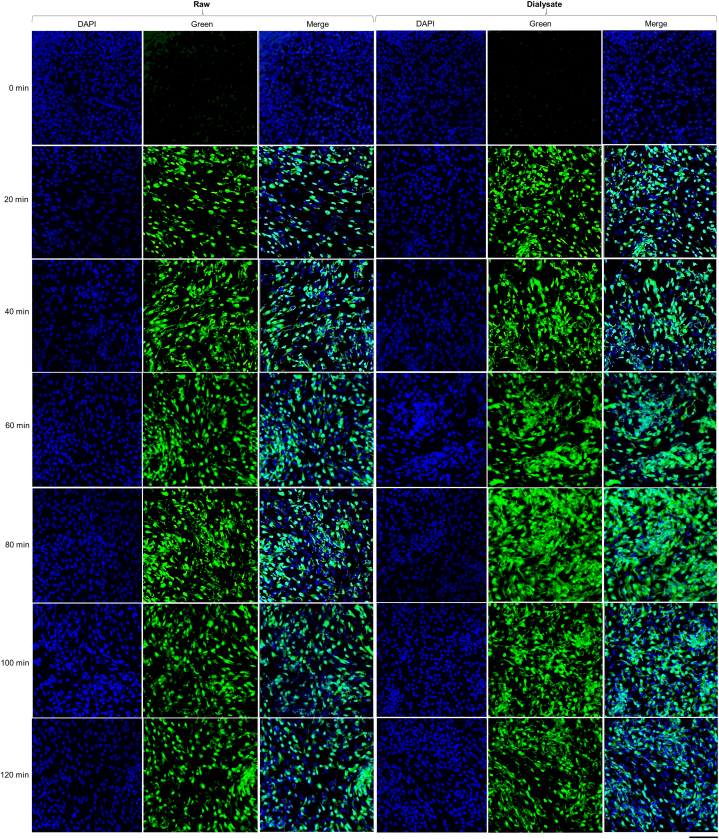
Represents the fluorescence images of CHO cells at different washout durations for 250 μg/mL of MMK raw and 250 μg/mL equivalent of MMK dialysate is shown using Confocal microscopy at 20× magnification.

Similarly, In Fig. 9C, the calcium Response % of MMK raw started to increase sharply from the 40th minute of washout period. From all the tested concentrations of MMK raw, 250 μg/ml was best able to prevent the intracellular cytosolic calcium. In Fig. 9D, MMK dialysate, the calcium response % started to increase from the 20th min and continuously increased till the 120th min. The MMK dialysate concentration which best inhibited intracellular cytosolic calcium was 250 μg/ml equivalent. Fig. 10, Fig. 11 represents the fluorescence images of CHO cells at 20× magnification after treatment with 250 μg/ml of HK raw and dialysate (Fig. 10) and MMK raw and dialysate (Fig. 11). The images were captured using confocal microscope.

## 5 Discussion

In this present experiment, HK and MMK were tested for anti-allergic activities through H1 receptor. Both the formulations prevented EB dye leakage in histamine and C 48/80 challenged mice. Both the formulations attenuated the number of sneezes and blood eosinophilic count in guineapigs. Further, they also decreased plasma histamine level in C 48/80 challenged mice. A decrease in intracellular cytosolic calcium level was exhibited by both the ayurvedic polyherbal drug formulations (HK and MMK) in CHO cells.

During allergic inflammation activated mast cells release histamine. This causes hyperpermeability in the blood vessels. Vascular hyperpermeability is linked to allergic symptoms like conjunctivitis, nasal congestion and urticaria. Understanding the mechanism behind how histamine causes vascular hyperpermeability might lead to new ways to treat allergic diseases. According to some scientific reports, histamine causes the breakdown of the endothelial cell barrier. Although many of these studies are conducted in *in vitro* systems, the underlying causes or mechanisms involved in *invivo* systems remain unclear. Histamine-induced increased blood flow causes hyperpermeability due to the liberation of NO and endothelial barrier disruption. Nowadays, vasoconstrictors are used in the treatment of rhinitis [25]. Our findings corroborate the usefulness of HK and MMK and suggest that they might be useful in treating various allergic symptoms.

As per published reports, leukotrienes primarily increased vascular permeability. Eosinophils are the primary originators of the cysteinyl leukotrienes LTC4/D4/E4, which are lipid byproducts that have significant functions in asthma and other types of allergic inflammation [26,27]. The vessel walls and smooth muscle layer of the venule are much more delicate than that of an artery or large vein and are permeable due to structural weakness. Structural weakness is the cause of less expression of adhesion protein. The integrity of the epithelial barrier is important for blood vessel permeability [28]. A Study suggests that the endothelial H1 receptor is functionally important in histamine-induced vascular leakage [29]. Inflammation is a clinical manifestation of an allergic reaction. Histamine causes vasodilation followed by flare. Endothelial cells are mural cells of blood vessels, especially capillary or end vessels. At end vessels, epithelial cells are connected with adhesion molecules like VE-Cadherin [30]. The presence of histamine makes detachment of adhesion molecules and plasma get leaked in extravascular space. In case of allergic conditions end vessel dilation causes redness under the skin.

Fluid accumulation and edema can also result from tissue insult directly or indirectly related to histaminergic response [31]. An *invitro* assay involving hypotonic solution induced goat RBC membrane disruption was conducted to determine the toxicity of HK and MMK on mammalian cell membrane. Both HK and MMK were observed to be nontoxic to mammalian cell membrane as was evident in the findings with the goat RBC experiment [Fig. 8].

The dye leakage test can be related to the membrane stabilization test (goat RBC) and indicates that HK and MMK had no role in plasma extravasation. The dye leakage test was conducted using two distinct principles. Primarily, through external histamine incorporation and secondly through a potent mast cell degranulator i.e. C 48/80 by provoking intrinsic histamine release. In both cases, dye leakage was prominent as described in our result [Fig. 1, Fig. 2].

When HK and MMK were given orally to the mice, a decreased amount of dye leaching out (Fig. 1, Fig. 2) (spectrophotometrically) was observed, which shows that these two formulations might diminish the activity of histamine. Externally [Fig. 1], HK showed greater ability than MMK in preventing the dye leakage while the reverse was observed i.e. dye leakage caused due to degranulation of mast cell by C 48/80 [Fig. 2].

In another study with guineapigs it was found that HK and MMK were able to decrease the number of sneezes [although 50 % of the population showed higher suppression of number of sneezes while others didn't] [Fig. 4]. The histamine chamber experiment (histamine sprayed in histamine chamber) involving guineapigs [Fig. 4] and dye leakage tests [ Fig. 1, Fig. 2] indicate the presence of histamine externally, extrinsicly (histamine administration) and intrinsicly (C 48/80) does not promote histaminergic responses when treated with HK and MMK indicating anti-allergic effect.

Furthermore, a study was conducted to measure the number of eosinophils in guinea pig's blood where before exposure to external histamine the test agents were administered. Treatment with both HK and MMK significantly decreased (p < 0.05) the blood eosinophil count. Histamine being a potent mediator of inflammation can stimulate the activation and production of eosinophils through H_1_ receptor mediated superoxide ions generation [32]. Eosinophilia is a hallmark of allergic inflammation. The sprayed histamine on guineapigs might have triggered inflammation which further released more histamine from mast cells, causing eosinophil activation and proliferation. Further, the activated eosinophils can attract more mast cells to the site of inflammation, generating a vicious cycle that amplifies the allergic response. Based on our experiment, it is evident that HK and MMK might have inactivated the H_1_ receptor in histamine exposed mice. Moreover, these ayurvedic formulations may also have stabilized mast cells in compound 48/80 induced mice. Therefore, it is quite reasonable to believe that these ayurvedic formulations might have played an important role in reducing the number of blood eosinophils in histamine sprayed guineapigs which can be co-related with the decreased sneezing rate in the same [Fig. 5].

The observed reduced plasma histamine (measured using LC-ESI-MS/MS) after C 48/80 challenge in the HK_48/80_ and MMK_48/80_ groups [Fig. 6, Fig. 7] and the decrease in EB dye leakage in the HK_48/80_ and MMK_48/80_ group [Fig. 2] indicate that the HK and MMK may probably prevent mast cell degranulation. Mast cell degranulation is a key step in the release of histamine [33]. From the above-mentioned observations, HK and MMK appear to prevent direct histamine release in the plasma as well as stabilize the mast cell from undergoing degranulation and subsequently releasing histamine. From the observed results, HK and MMK activity may be related to their mast cell stabilising effect.

Among the many factors that govern histamine release calcium-mediated calcium release from ER where Calcium is the second messenger, is the most important event which ultimately culminates in exocytosis. Finally, this exocytosis is responsible for histamine release from mast cells [34,35].

Thus, intracellular cytosolic calcium seems to play a significant part in ensuring either histamine release or its inhibition. Moreover, the histamine release process is primarily mediated through calcium involving phospholipase C and phospholipase A2 pathways activation [36,37]. Initially released Ca^2+^ from ER binds to the ryanodine receptors (RyRs) on the ER which again triggers more release of Ca^2+^ from it. HK and MMK both in raw formulation and their dialysate, were studied for intracellular calcium release in histamine-challenged CHO cells [Fig. 9].

The intracellular calcium assay in CHO cells is presented in Fig. 9. In this study, the histaminergic response has been presented as a percentage abundance of calcium ions present within the cell. This finding can also be correlated with histamine binding to H1 receptors resulting in subsequent liberation of Ca^2+^ from ER. The presence of HK [Fig. 9A and B], decreased the appearance of intracellular cytosolic calcium, even after the addition of histamine at all-time points with subsequent washouts up to 40th min (for HK raw) and 20th mins (for HK dialysate) indicating non-responsiveness of H1 receptor-mediated Ca^2+^ release. This probably indicates HK both raw and in dialysate form, hindered histamine to release intracellular cytosolic calcium via H1 receptor in CHO cells. Similar findings were observed in the MMK both raw up to 40th minute [Fig. 9C] and in dialysate form up to 20th minute [Fig. 9D].

Further, in the same study after the 60th minute histamine-mediated Ca^2+^ release was observed to be almost saturated till the end point. This might be due to the desensitization of histamine receptors owing to the presence of HK and MMK both in raw and in dialysate form.

As per our observation, both HK and MMK in raw form showed more inhibition for histamine-mediated Ca^2+^ release as compared to their dialysate form. Among many probable reasons behind this phenomenon, it can be due to the synergistic/holistic effect of multiple components of these clinically used ayurvedic polyherbal drug formulations. However, the dialysate form of both HK and MMK, showed comparatively better dose-related response. It will be important to mention that the dialysate form (involving 12-14kD membrane) of these preparations corresponds to the behaviour of these formulations in plasma following oral administration of HK and MMK.

Therefore, HK and MMK both raw/dialysate indicate H1 receptor inactivation resulting in decreased intracellular cytosolic Ca^2+^ appearance indicating prevention of exocytosis of various cells like mast cells. It will be relevant to mention here that these two formulations significantly inhibited C 48/80 mediated dye leakage with a simultaneous reduction in plasma histamine in mice, which has already been mentioned earlier in this text.

The traditional use of HK and MMK has been mentioned above [9,10]. There are many plant components present in the two ayurvedic formulations. These include *Curcuma longa, Zingiber officinale, and Piper nigrum* in HK, and *Rubia cordifolia*, *Piper longum*, and *Tinospora cordifolia* in MMK, as well as additional plant components. The scientific reports indicated a substantial anti-allergic effect of the plant components stated above [[38], [39], [40], [41], [42], [43]].

The aforementioned investigations demonstrated that the experimental ayurvedic formulations provided protection against histamine-induced allergy manifestation by decreasing capillary permeability, stabilising mast cells, and alleviating allergic rhinitis. The aforementioned observations further motivated the utilisation of a Ca^2+^ release assay. HK and MMK exhibited a dose-dependent suppression of histamine-induced calcium release in CHO cells. HK and MMK appears to protect against histamine mediated allergy modulating intracellular cytosolic calcium.

In Ayurveda, HK has been used to manage Vataja Pratishyaya, a terminology used in their system which can be co-related to allergic rhinitis in modern medicine [10,44]. MMK has also been reported to act against some skin diseases as mentioned in ayurvedic literature [11]. The above mentioned ayurvedic literature led us to form a hypothesis that both HK and MMK might play a role in alleviating histamine mediated allergic rhinitis. Thus, based on our experiment, this reverse pharmacological study appears to validate the rationality and conceived hypothesis for the clinical use of HK and MMK in histamine mediated allergic rhinitis, within the ambit of our study's limitations.

## 6 Conclusion

In our experiment, we have introduced administration of histamine externally mimicking sudden clinical anaphylactic condition. We have also arranged for the intrinsic release of mast cell histamine with compound 48/80. During the course of the investigation guineapig sneezing in histamine chamber was induced to mimic rhinitis. In the experiment, the standard drug pheniramine maleate was used for the suppression of histamine-mediated H1 receptor activity. However, sodium chromoglycate was used as a standard mast cell stabilizer. In all the above-mentioned experiments investigational ayurvedic preparation showed protection against histamine-induced allergic manifestation which involved increased capillary permeability, mast cell stabilization, and allergic rhinitis as compared to allopathic medicine pheneramine. Observations mentioned so far prompted to involve Ca^2+^ release assay. Interestingly, enough HK & MMK showed dose-dependant inhibition of histamine-induced Ca^2+^ release in CHO cells. Finally, besides antiallergic activity of these two clinically used ayurvedic preparations it was also found to be nontoxic to mammalian cell membrane as was evident in the findings with the goat RBC experiment. The component plants of these two ayurvedic formulations e.g. *Curcuma longa, Zingiber officinale* and *Piper nigrum* component of HK and *Rubia Cordifolia, Piper longum* and *Tinospora cordifolia* component of MMK along with other plant components have been reported earlier. Report revealed significant anti-allergic action/potential of the above-mentioned plant components. It will be important to mention that report suggests that polyherbal combinations are often beneficial from the therapeutic point of view as compared to that of the individual plant component [8]. Therefore, this reverse pharmacological investigation probably justifies the clinical use of HK and MMK within the limitation of our experiment.

## Ethics approval

The animal study protocol was approved by the Institutional Animal Ethics Committee (IAEC) of Dept. of Pharmaceutical Technology, Jadavpur University, through project proposal no. JU/IAEC-22/21.

## CRediT authorship contribution statement

**Rudranil Bhowmik:** Writing – original draft, Methodology, Formal analysis, Conceptualization. **Md Adil Shaharyar:** Writing – original draft, Methodology, Data curation. **Mahibub Mahamadsa Kanakal:** Writing – review & editing, Funding acquisition. **Arnab Sarkar:** Writing – original draft, Software, Resources, Data curation. **Syeda Ayesha Farhana:** Writing – review & editing, Funding acquisition. **Shalam M. Hussain:** Validation, Methodology. **Abdullah Khan:** Writing – review & editing, Validation. **Pallab Mandal:** Visualization, Validation, Formal analysis. **S. Roshan:** Writing – review & editing, Visualization. **Achintya Mitra:** Writing – review & editing, Validation, Formal analysis. **Sanmoy Karmakar:** Writing – review & editing, Supervision, Funding acquisition, Conceptualization.

## Declaration of competing interest

The authors declare that they have no known competing financial interests or personal relationships that could have appeared to influence the work reported in this paper.

## Abbreviations

b.w.: body weight
C 48/80: Compound 48/80
EB: Evans blue
HBSS: Hank's Balanced Salt Solution
HK: Haridra Khanda
i.v.: intravenous
LC: liquid chromatography
MMK: Manjishthadi Kwatham (brihat)
MRM: Multiple reaction monitoring
p.o.: per os
QTRAP: quadruple ion trap.
